# A Highly Polymorphic Copy Number Variant in the *NSF* Gene is Associated with Cocaine Dependence

**DOI:** 10.1038/srep31033

**Published:** 2016-08-08

**Authors:** Judit Cabana-Domínguez, Carlos Roncero, Lara Grau-López, Laia Rodríguez-Cintas, Carmen Barral, Alfonso C. Abad, Galina Erikson, Nathan E. Wineinger, Bàrbara Torrico, Concepció Arenas, Miquel Casas, Marta Ribasés, Bru Cormand, Noèlia Fernàndez-Castillo

**Affiliations:** 1 Departament de Genètica, Microbiologia i Estadística, Facultat de Biologia, Universitat de Barcelona, Barcelona, Catalonia, Spain; 2 Centro de Investigación Biomédica en Red de Enfermedades Raras (CIBERER), Instituto de Salud Carlos III, Spain; 3 Institut de Biomedicina de la Universitat de Barcelona (IBUB), Barcelona, Catalonia, Spain; 4 Institut de Recerca Pediàtrica Hospital Sant Joan de Déu, Esplugues, Barcelona, Catalonia, Spain; 5 Department of Psychiatry, Hospital Universitari Vall d’Hebron, Barcelona, Catalonia, Spain; 6 Addiction and Dual Diagnosis Unit Clinic Vall Hebron, Psychiatric Services, Hospital Universitari Vall d’Hebron-ASPB, Barcelona, Catalonia, Spain; 7 Psychiatric Department and Legal Medicine. Universitat Autònoma de Barcelona, Catalonia, Spain; 8 Centro de Investigación Biomédica en Red de Salud Mental (CIBERSAM), Instituto de Salud Carlos III, Spain; 9 Scripps Translational Science Institute, La Jolla, CA, United States of America; 10 Psychiatric Genetics Unit, Hospital Universitari Vall d’Hebron, Universitat Autònoma de Barcelona, Barcelona, Catalonia, Spain

## Abstract

Cocaine dependence is a complex psychiatric disorder involving both genetic and environmental factors. Several neurotransmitter systems mediate cocaine’s effects, dependence and relapse, being the components of the neurotransmitter release machinery good candidates for the disorder. Previously, we identified a risk haplotype for cocaine dependence in the *NSF* gene, encoding the protein N-Ethylmaleimide-Sensitive Factor essential for synaptic vesicle turnover. Here we examined the possible contribution to cocaine dependence of a large copy number variant (CNV) that encompasses part of the *NSF* gene. We performed a case-control association study in a discovery sample (359 cases and 356 controls) and identified an association between cocaine dependence and the CNV (*P* = 0.013), that was confirmed in the replication sample (508 cases and 569 controls, *P* = 7.1e-03) and in a pooled analysis (*P* = 1.8e-04), with an over-representation of low number of copies in cases. Subsequently, we studied the functional impact of the CNV on gene expression and found that the levels of two *NSF* transcripts were significantly increased in peripheral blood mononuclear cells (PBMC) along with the number of copies of the CNV. These results, together with a previous study from our group, support the role of *NSF* in the susceptibility to cocaine dependence.

Cocaine dependence is a complex psychiatric disorder that results from the interaction between genetic and environmental factors, with an estimated heritability of 65–79%^1^,^2^,^3^. Cocaine inhibits dopamine, serotonin and norepinephrine reuptake by binding to their transporters (DAT1, SERT and NET, respectively), increasing their levels at the synaptic cleft^4^,^5^. Also, other neurotransmitter pathways participate in cocaine dependence, withdrawal or relapse, including the glutamatergic, GABAergic and endocannabinoid systems^6^,^7^,^8^,^9^,^10^,^11^,^12^. Their involvement in mediating cocaine effects and dependence makes the molecular machinery that controls neurotransmitter release to the synaptic cleft a strong candidate for participating in the development of cocaine dependence. As part of this complex process that involves many different proteins, the N-ethylmaleimide sensitive factor (NSF) plays an essential role in the synaptic vesicle turnover. NSF is responsible for the recycling of Soluble NSF Attachment Protein Receptor (SNARE) complexes, preventing their accumulation and ensuring the maintenance of intracellular membrane trafficking^13^.

Previous genetic evidence supports the contribution of single-nucleotide polymorphisms (SNPs) in the *NSF* gene to cocaine addiction, where a two-SNPs risk haplotype was associated with a rapid development of cocaine dependence, shortly after the first cocaine use^14^. To date, association studies with SNPs have unraveled only a small fraction of the estimated heritability for cocaine dependence. Thus, exploring structural variation may help to capture part of the missing heritability underlying this complex disorder^15^. Interestingly, a large copy number variant (CNV) of about 734 kb encompasses the first 13 exons of the *NSF* gene (including two additional genes, *LRRC37A2* and *ARL17A*) and ranges from 2 to 6 copies per individual (1 to 3 haploid copies)^16^. In the European population, about 72% of individuals have more than two copies. This CNV is located immediately distal from the *MAPT* locus at 17q21.31, which was previously found to be subjected to positive selection and rapid evolutionary turnover, and associated with neurological disorders^17^.

Since CNVs are common forms of genetic variation that often impact gene expression and may have a greater effect on transcriptional regulation than that attributed to SNPs^18^,^19^,^20^, we hypothesize that the CNV spanning *NSF* has an influence on the expression of the gene and may contribute to cocaine dependence. To our knowledge, association between CNVs and drug dependence has been explored mainly for alcohol or opioid dependence^21^,^22^,^23^,^24^, but no study has investigated cocaine dependence so far. To address this issue, we explored the role of the CNV encompassing *NSF* in cocaine addiction through a two stage case-control association study with cocaine dependence. Subsequently, we studied the linkage disequilibrium (LD) between the previous associated SNPs and the CNV, as well as the impact of this CNV on *NSF* expression.

## Results

**Figure 1:**
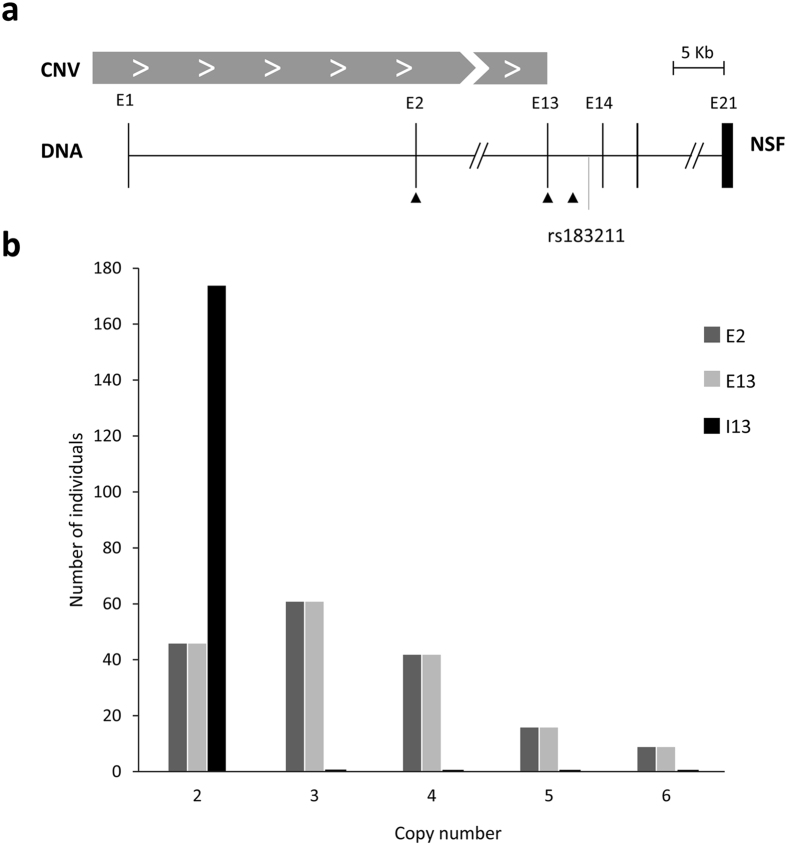
Delimitation of the CNV region. (**a**) Representation of the 3′ end of the CNV spanning the *NSF* gene. The CNV is indicated with a grey box, exons with black boxes, and triangles show the position of primers used in qRT-PCR assays. The location of SNP rs183211 is indicated. (**b**) Number of individuals grouped by copies of the *NSF* CNV in exons 2 and 13 and in intron 13.

**Figure 2:**
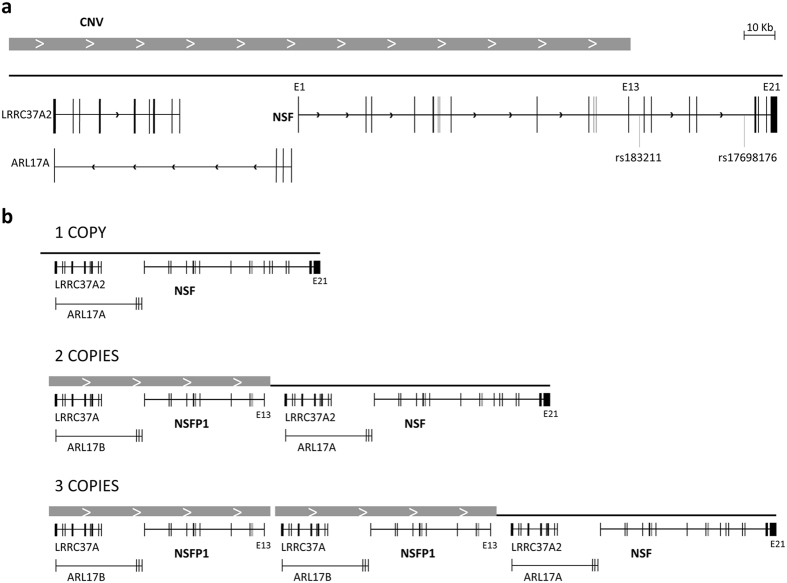
Schematic representation of the CNV region. (**a**) Representation of genes *NSF* (ENSG00000073969), *ARL17A* (ENSG00000185829) and *LRRC37A2* (ENSG00000238083). Exons are indicated with black boxes, CNV is indicated with a grey box. The location of the two SNPs previously associated with cocaine dependence is indicated. (**b**) Schema of genetic context of the three possible alleles of the CNV in this region: 1, 2 or 3 repeats.

A CNV encompassing part of the *NSF* gene was previously reported^16^. According to published data and information in the Ensembl database (www.ensembl.org), it spans the first 13 exons of the gene. We refined the distal end of the CNV in our population by amplifying two *NSF* regions by quantitative real-time PCR (qRT-PCR), one in exon 13 and another one in intron 13 (Fig. 1a), and comparing the results with an internal CNV control in exon 2. All subjects were confirmed to bear only two copies of the DNA segment in intron 13, and a variable (and identical) number of copies in exons 13 and 2 (Fig. 1b), confirming that the CNV encompasses the first 13 exons of the gene in all 174 individuals assessed, ranging from 2 to 6 copies per diploid genome (Fig. 2).

In order to explore whether the *NSF* CNV was associated with cocaine dependence, we performed a case-control association study. The comparison of the number of copies of the CNV between 359 cocaine-dependent patients and 356 controls showed significant differences between groups (*P* = 0.036, Supplementary Table S1). When we subdivided subjects into ‘low number of copies of the CNV’ (2 and 3) and ‘high number of copies’ (4, 5 and 6), we identified a significant overrepresentation of low number of copies in cocaine-dependent patients (72% *vs* 63%; *P* = 0.013; OR = 1.51, CI = [1.10–2.07]). This overrepresentation was replicated in an independent sample (69% *vs* 63%; *P* = 7.1e-03; OR = 1.42, CI = [1.10–1.83]). The pooled analysis of both the discovery and replication samples also rendered statistically significant results (71% *vs* 62%; *P* = 1.8e-04; OR = 1.459, CI = [1.20 –1.78]), which remained significant after adjusting by age (*P*_adj_ = 3.1e-03), with a higher frequency of subjects carrying 2 or 3 copies of the *NSF* CNV in the group of cocaine-dependent subjects when compared to controls (Table 1).

**Figure 3:**
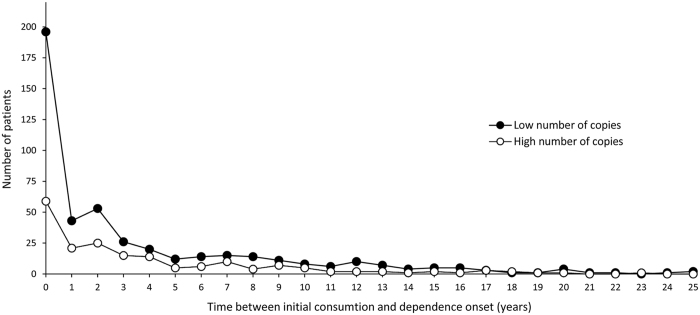
Time between initial consumption and onset of cocaine dependence (years) in individuals with low (2 or 3) and high (4, 5 or 6) number of copies of the *NSF* CNV.

Then, we observed that individuals with low number of copies showed an earlier dependence onset than individuals with high number of copies (*P* = 0.042). The main differences were observed in the group of patients that develop cocaine dependence within a year after the first drug use (Fig. 3). When we subdivided subjects in two subgroups, early (<1 year) and late (≥1 year) dependence onset, we identified an overrepresentation of individuals with low number of copies in the group of patients showing early regular cocaine consumption (*P* = 5e-03; OR = 1.62, CI = [1.13–2.32]).

Previously, a SNP located in intron 13 of the *NSF* gene, rs183211, was associated with cocaine dependence either as a single marker or as part of a risk haplotype with rs17698176 (intron 17; Fig. 2a)^14^, also associated with an early onset of cocaine dependence. These two SNPs are located distal from the CNV according to our above experiments. Thus, we studied whether the associations between cocaine dependence and either the CNV or the two SNPs in the risk haplotype at *NSF* were related. To do so, we estimated SNP-CNV haplotype frequencies using an expectation-maximization (EM) algorithm, and subsequently assessed LD between the CNV and rs183211 (Supplementary Table S2) and the CNV and the rs183211-rs17698176 risk haplotype (Table 2). We found that the rs183211G risk allele was associated with low number of copies at the CNV (*P* = 6e-12) as it was in moderate LD with one copy (r^2^ = 0.30). Furthermore, similar results were obtained for the risk haplotype: the rs183211G-rs17698176T haplotype allele was associated with low copy number of the CNV (*P* < 2.2e-16) as this previously reported risk haplotype was in LD with one copy (r^2^ = 0.61).

**Figure 4:**
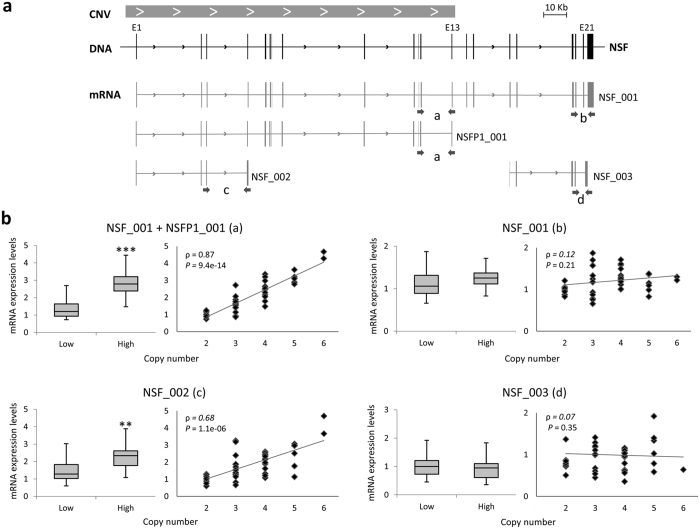
Correlation between the number of copies of the CNV and gene expression. (**a**) Structure of transcripts detected by qRT-PCR assay. CNV is indicated with a grey box, exons are indicated with boxes in black for the *NSF* gene (ENSG00000073969) and in grey for *NSF* transcripts: NSF_001 (ENST00000398238), NSFP1_001 (ENST00000570034), NSF_002 (ENST00000486366) and NSF_003 (ENST00000465370). Arrows indicate the position of qRT-PCR primers sets used to detect the different transcripts: “a” for NSF_001 + NSFP_001, “b” for NSF_001, “c” for NSF_002 and “d” for NSF_003. (**b**) Blox plot for mRNA expression levels of each transcript in individuals with low and high number of copies. Linear regression showing the correlation between number of copies and transcript expression levels. Spearman’s rank correlation coefficients (ρ) are shown inside the graphs. (^**^P < 0.001; ^***^P < 0.0001).

The two intronic SNPs previously reported to be associated with cocaine dependence, rs183211 and rs17698176, had no predicted functional effects according to the software SNP Function Prediction (snpinfo.niehs.nih.gov/snpinfo/snpfunc.htm). As we found association between the risk haploallele rs183211G-rs17698176T and low number of CNV copies, we subsequently explored the potential impact of the CNV on the expression of the *NSF* gene. Expression levels for NSF_001 (full length transcript), NSF_002 (including the first four exons) and NSF_003 (including the last five exons) could be determined in PBMCs. Moreover, *NSFP1*, that spans the first 13 exons of the gene and seems to be a pseudogene that results from the CNV dynamics, encodes another transcript named NSFP1_001 (Fig. 2b). We analyzed the expression levels of this last transcript with an assay that also detects one of the gene products, NSF_001 (Fig. 4a, primer pair ‘a’). As shown in Fig. 4b, the expression levels of transcripts NSF_001 + NSFP1_001 and NSF_002 were significantly increased along with the number of copies of the CNV (*P* = 4.3e-06 and *P* = 8.9e-05, respectively). Similar results were observed when the individuals were grouped into low and high number of copies, with transcript levels increased by 2.1-fold and 1.5-fold for NSF_001 + NSFP1_001 and NSF_002, respectively, in the group of high number of copies as compared to the group of low number of copies (*P* = 8.5e-09 and *P* = 3.7e-04, respectively). Finally, we assessed the linearity of this correlation and found a significant association between the number of copies of the CNV and the level of transcripts NSF_001 + NSFP1_001 (ρ = 0.87, *P* = 9.4e-14) and NSF_002 (ρ = 0.68, *P* = 1.18e-06). As expected, no significant changes were observed for transcripts NSF_001 and NSF_003, as they are encoded by genomic regions outside the CNV (Fig. 4b).

## Discussion

Many studies have been performed to identify SNP variants associated with drug dependence and, even though some of them have reported significant findings, only a small fraction of the estimated heritability of this complex phenotype can be explained by them. Methodological limitations may account in part for the missing heritability, but other sources of genetic variation, like copy number variants, could also contribute to the genetic landscape of the disorder^15^. In this study, we identified a significant association between cocaine dependence and a CNV spanning the first 13 exons the *NSF* gene. To our knowledge, this is the first study that associates a CNV with cocaine dependence.

In a previous study, our group identified an association between a quick transition from cocaine use to dependence and a risk haplotype (rs183211G-rs17698176T) in the *NSF* gene. Furthermore, a large polymorphic CNV partially overlapping the *NSF* gene has been described^16^. We thus hypothesized that the *NSF* CNV could contribute to the susceptibility to cocaine dependence by altering gene expression. First, we performed a case-control association study and found a significant association between this CNV and cocaine dependence, with an overrepresentation of individuals with low number of copies in cocaine patients. Then, we observed that a low number of CNV copies correlated with a rapid development of dependence, and that the CNV and the previously described risk haplotype were in LD. Furthermore, we observed an effect of the CNV on *NSF* expression, identifying a positive correlation between the number of copies of the CNV and the levels of two transcripts, NSF_002 and NSFP1_001, both encoded by the segment of the gene that is included in the CNV. Since we had no access to brain samples, gene expression was assessed in samples obtained from PBMC, which may differ from brain *NSF* expression. Some studies, however, reveal a correlation in gene expression levels between peripheral blood and brain^25^.

In addition, we have characterized the 3′ end of the CNV, located within the *NSF* gene, and confirmed that the two disease-associated SNPs are located outside the CNV but in moderate LD with it. In consequence, the association identified previously, which was detected under the assumption of diploidy for the SNP variants, was calculated correctly and suggests that the two intronic SNPs may tag the *NSF* CNV.

CNVs play an important role in the generation of genetic diversity and so in human evolution. They have also been shown to have phenotypic effects, including susceptibility to disease, depending on whether or not dosage-sensitive genes or regulatory sequences are affected by the rearrangement^26^,^27^. Many genes involved in brain development are enriched in CNVs^28^, the biological importance as well as the evolutionary conservation of these genes suggest that they could have phenotype implications and might contribute to the susceptibility to complex neuropsychiatric disorders^29^,^30^. Genome-wide studies performed in drug abuse have unraveled a number of CNVs possibly contributing to drug dependence. For example, a CNV at 11q14.2 was found associated with variations in brain volume and drinking behaviour^21^, and losses at 22q13.1 seem to increase the response to alcohol in the precuneus, a region previously associated with severity of alcohol dependence (AD)^22^. Furthermore, a GWAS assessing common CNVs in AD in a Korean cohort identified losses at 20q13.33 as protective factors for AD^23^. Recently, a GWAS performed in opioid dependence (OD) identified many CNVs which could contribute to resistance or susceptibility to OD, three of them genome-wide significant^24^. These findings suggest that CNVs may have an important role in drug dependence. However, to our knowledge, the present study is the first one performed in cocaine dependence considering CNVs, and obtaining significant results.

The *NSF* gene encodes the hexameric ATPase N-ethylmaleimide-sensitive factor, which plays an essential role in the synaptic vesicle turnover, modulating the kinetics of neurotransmitters and the integrative properties of synapses^31^,^32^,^33^. NSF operates in the recycling of the SNARE complexes by preventing the accumulation of cis-complexes and assuring a sufficient amount of free SNARE for the maintenance of the intracellular membrane trafficking^13^. Furthermore, several studies suggest that NSF might modulate the function of several neurotransmitters in the central nervous system by interacting with their receptors, such as the glutamatergic alpha-amino-3-hydroxy-5-methylisoxazole-4-propionic acid receptor (AMPA), the beta-2 adrenergic receptor (β2-AR), the dopamine receptors (more strongly D1 and D5), the adrenomedulin (AM) receptor, the γ-amino-butyric acid receptor (GABA_A_) and the serotonin transporter (SERT)^34^,^35^,^36^,^37^,^38^,^39^,^40^,^41^,^42^,^43^,^44^,^45^,^46^. Additionally, a recent study identified interactions between NSF and BLOC-1 (biogenesis of lysosome-related organelles complex 1), which participate in synaptic vesicle assembly, neurotransmission and plasticity, and this interaction modulates synaptic plasticity^47^.

Interestingly, a recent study showed that withdrawal from repeated cocaine exposure increased interactions between NSF and glutamate receptor 2 (GluR2), and this complex acts as a negative regulator of the expression of behavioral sensitization in rats^48^. Moreover, the *NSF* gene was found significantly down-regulated in postmortem hippocampus of cocaine addicts samples, which is consistent with our results showing an overrepresentation of low number of copies (which correlates with lower expression) in our sample of cocaine dependent patients^49^. Furthermore, *NSF* decrease correlates with a robust down-regulation of *GABBR1* (Gamma-aminobutyric acid type B receptor subunit 1), a subunit of GABAB receptor, which could be used as a predictor for increased risk for addiction^49^. And in autistic patients, *NSF* expression was also found significantly reduced in lymphocytes and tended to be reduced in post-mortem brains, correlating with the severity of clinical traits^46^.

The CNV spanning the *NSF* gene was previously associated with elevated peak antibody response to the AVA-Biothrax vaccine, suggesting that this CNV has an effect on the expression of the genes in this region (*NSF*, *ARL17* and *LRRC37A*)^50^. We have shown that a CNV spanning the *NSF* gene produces changes in the expression levels of some *NSF* transcripts, which could have an effect on the availability of neurotransmitter vesicles (loaded with e.g. dopamine, essential in the reward system) and its turnover, producing changes in the neurotransmitter release to the synaptic cleft. This would be in line with the “reward deficiency syndrome”, which hypothesizes that hypodopaminergic activity predisposes to cocaine dependence^51^. Although DA neurotransmission has been established as a central mediator in cocaine effects^52^,^53^,^54^,^55^, other neurotransmitters systems, such as the glutamatergic, GABAergic and endocannabinoid ones, also dependent on NSF activity, are very important and both association studies and animal models support their role in cocaine dependence^56^,^57^,^58^,^59^,^60^,^61^.

The present case-control association study raises several methodological issues: (i) sample heterogeneity might alter the results of association studies. To minimize the risks we considered sex-matched Caucasian patients and controls from Spain, all of them recruited in a small geographical area around Barcelona and evaluated following a common clinical assessment; (ii) cocaine dependence was not discarded in the control sample. However, this problem, which tends to balance allele frequencies in cases and controls, would increase the probability of false negative results, which is not the case in the present study; (iii) qRT-PCR assays are widely used to validate CNVs because of its specificity and reproducibility, even though in some cases the results may fall between integers (e.g. copy number measuring 1.4), making interpretation difficult. Ambiguous results were discarded from our study in order to ensure a correct assignation of the number of copies in all cases; (iv) Hardy-Weinberg equilibrium (HWE) could not be tested in our samples since our genotyping strategy did not allow establishing phases. However, the effects of CNVs are usually related to genetic dose, therefore what matters is the total number of copies and not the phased genotype; (v) considering the number of CNV copies as low (2 and 3) or high (4, 5 and 6) is somehow arbitrary (Supplementary Table S1). In any case, if we consider individuals with 4 number of copies into the ‘low’ group, the association remains statistically significant (*P* = 8.3e-04); (vi) we assessed the effect of the CNV on gene expression in RNA from PBMC, which may differ from expression in the brain; (vi) it is important to note that other genes in addition to *NSF* lie in the CNV and could also be contributing to cocaine dependence predisposition.

To conclude, we found a significant association between cocaine dependence and a CNV spanning the *NSF* gene. Functional studies revealed that the levels of two transcripts in blood, NSFP_001 and NSF_002, correlate with the number of copies of the CNV, suggesting that this CNV might have a functional impact on gene expression. These results, together with previous studies by our group^14^, suggest that *NSF* contributes to the genetic predisposition to cocaine dependence and, especially, to an early onset of dependence.

## Materials and Methods

### Delimitation of the copy number variation

The extension of the CNV spanning the *NSF* gene was ascertained in 174 individuals (89 controls and 85 cases). Detailed information about the subjects is provided in the ‘Association study’ section.

The number of copies at the CNV region and outside was assessed by qRT-PCR considering three regions of the *NSF* gene: exon 2, exon 13 and intron 13 (the latter located 102 bp 5′ from SNP rs183211, previously associated with cocaine dependence) to narrow down the CNV extension. The albumin gene (*ALB*) was used as a diploid control. Primer sequences are available in Supplementary Table S3. QRT-PCR assays were performed as follows: 7.5 μl of LightCycler^®^ 480 SYBR Green I Master (Roche Life Sciences, Branford, CT, USA), 400 nM of each primer, 10 ng of DNA and PCR-grade water up to 15 μl. The qRT-PCR conditions were: 10 min at 95 °C, 40 cycles of 95 °C for 15 s and 60 °C for 1 min. Specificity was ascertained after completion of the amplification in the presence of SYBR Green by the melting procedure (ramping from +65 °C to +97 °C, rising the temperature by 2.2 °C at every step with 2-seconds intervals). QRT-PCR experiments were performed using the LightCycler^®^ 480 System and results were analyzed with the LightCycler^®^ 480 Software, Version 1.5 (Roche Life Science, Branford, CT, USA). Standard curves for all amplicons were constructed through triplicate qRT-PCR amplifications with serial dilutions (factor of 2.5) of the DNA template and detection with SYBR Green. The final amount of template in the curve ranged from 300 ng to 3.14e-02 ng for DNA. Standard curves were used to determine the linearity (*R*^2^) and the efficiency (E)^62^ of the qRT-PCR amplifications and all transcripts were amplified with an efficiency around 94% (E = 1.94) and *R*^2^ = 0.9964, without significant differences among them. Five HapMap subjects with known number of copies at the *NSF* CNV (NA18506, NA19239, NA19240, NA12156, NA18942) were used as a reference for 2, 3, 4, 5 and 6 copies, respectively^16^. Genomic samples of these individuals were obtained from the Coriell Institute for Medical Research (New Jersey, USA) and were included in all assays as an internal control. *NSF* gene copy number was calculated by the comparative Ct (ΔΔCt) method and all experiments displaying ambiguous results were discarded from the study.

### Association study

#### Subjects

The discovery sample consisted of 359 cocaine-dependent patients (mean age 35.5 ± 8.1 years, 81.3% males (n = 292)) and 356 controls (52.7 ± 16.8 years, 81.5% males (n = 290)). The replication sample consisted of 508 cocaine-dependent patients (mean age 35.7 ± 7.8 years, 79.9% males (n = 406)) and 569 controls (52.1 ± 16.9 years, 79.8% males (n = 454)). All patients were recruited and evaluated at the Psychiatry Department of the Hospital Universitari Vall d’Hebron (Barcelona, Spain) according to DSM-IV TR criteria (Diagnostic and Statistical Manual of Mental Disorders, 4^th^ ed., text revision) and the SCID (Structured Clinical Interview)^63^. A total of 75% of patients (n = 652) reported age at the initial consumption as well as age at dependence onset. All controls were recruited at the Blood and Tissues Bank of Barcelona. None of them had injected drugs intravenously. Patients and controls were Spanish, Caucasian and sex-matched. The study was approved by the ethics committee of our institution, the Institutional Review Board of the University of Barcelona (IRB00003099), and informed consent was obtained from all participants, in accordance with the Helsinki Declaration. Population stratification was previously discarded in our sample^64^.

#### DNA isolation and quantification

Genomic DNA was isolated from peripheral blood mononuclear cells (PBMC) using the salting-out method^65^. DNA concentrations were determined using NanoDrop ND-1000 Spectrophotometer (NanoDrop Technologies, Termo Fisher Scientific Inc., Wilmington, DE, USA).

#### Copy number assay

The number of copies of the CNV of each individual was assessed by qRT-PCR considering a region of exon 2 in the *NSF* gene. The albumin gene (*ALB*) was used as a diploid control. QRT-PCR conditions are described above. In all assays, samples with known number of copies at the *NSF* CNV were included, showing a concordance rate of 100%. All experiments displaying ambiguous results were not considered in the study.

#### Statistical analysis

The comparison of the number of copies of the CNV between cases and controls was performed using the Fisher’s exact test (two-tailed), considering the five possible copy number assignations per individual (from 2 to 6) and also a dichotomization into low (2 or 3 copies) and high (4, 5 or 6 copies) copy number. Age was considered as a covariate using binary logistic regression test. To analyze the effect of the number of copies of the CNV in the lapse between initial and regular consumption of cocaine, the comparison of medians was performed using the Mann-Whitney U non-parametric test as normality was rejected using Kolmgorov-Smirnov test. In addition, the time between initial consumption and onset of cocaine dependence was dichotomized intro early (<1 years) or late (≥1 years) dependence onset, and the comparison of the number of copies of the *NSF* CNV between these groups was performed with the Fisher’s exact test (one-tailed). Statistical analysis was performed using SPSS Statistics Version 22.0 software (IBM Corp. Released 2013. Armonk, NY).

### Linkage disequilibrium analysis

We estimated the allele and haplotype frequencies of the CNV and two *NSF* SNPs found previously associated with cocaine dependence (rs183211 and rs17698176)^14^. Genotypes for both SNPs and CNV were available for 47% of the sample used in the CNV association study (n = 407, 36.07 ± 9.5 years, 85.7% males). Traditional approaches to assess LD between SNPs and CNVs are insufficient for higher order CNVs as the phase of the CNV cannot be directly inferred. To address this issue, we developed an EM algorithm-based method to estimate SNP-CNV haplotype frequencies under an assumption of Hardy-Weinberg equilibrium between haplotypes. The approach is similar to an existing method^66^. The algorithm begins by seeding initial SNP-CNV haplotype frequencies according to known SNP-CNV genotype counts (e.g., an individual with a homozygote AA SNP genotype and 2 copies of the CNV would likely have two copies of a haplotype which included the A SNP allele and a single copy of the CNV). In the E-step, haplotype counts are calculated based on the estimated haplotype frequencies; followed by the M-step, which recalculates haplotype frequencies based on the estimated haplotype counts. This process is repeated in an iterative fashion until the haplotype frequencies become constant from one iteration to the next. In this study, the frequency estimates were constant to five decimal places by the twentieth iteration. To estimate these haplotype frequencies, genotype data for rs183211 and rs17698176 from the 407 individuals previously described was used. From the resulting haplotype frequencies, we then assessed linkage disequilibrium (r^2^) between rs183211 and low number of copies of the CNV (individuals with 2 and 3 copies). Furthermore, we likewise assessed LD between the rs183211-rs17698176 haplotype and low number of copies of the CNV.

### *NSF* expression assays

To correlate the number of copies of the CNV with *NSF* transcription levels, 45 Spanish, Caucasian, healthy volunteers (mean age 32.8 ± 11.8 years, 66.7% females (n = 30)) were recruited at the Genetics Department of the University of Barcelona (Barcelona, Spain). Genomic DNA was isolated as previously described. Total RNA was extracted from PBMC within 2 h after sampling using BD Vacutainer^®^ CPT^TM^ tubes (BD, Franklin Lakes, NJ, USA) and the High Pure RNA Isolation Kit (Roche Life Science, Branford, CT, USA). RNA was retro-transcribed to cDNA using the High Capacity cDNA Reverse Transcription Kit (Applied Biosystems, Foster City, CA, USA). Specific primers were designed to detect each of the eight *NSF* transcripts according to ENSEMBL, as well as *NSFP1*, a pseudogene that probably arose as a consequence of the CNV dynamics. Three transcripts could be amplified specifically: NSF_001 (primers set ‘b’ in Fig. 4a), NSF_002 (primers set ‘c’) and NSF_003 (primers set ‘d’) (ENST00000398238, ENST00000486366 and ENST00000465370, respectively, according to Ensembl release 83). For NSFP1_001 (ENST00000570034.1) we could not design specific primers since it overlaps with NSF_001; for this reason we designed a qRT-PCR assay to detect simultaneously both transcripts (primers set ‘a’). Glyceraldehyde-3-phosphate dehydrogenase *(GAPDH)* and hypoxanthine phosphoribosyltransferase *(HPRT1*) were used to normalize the relative amounts of mRNA. QRT-PCR assays were performed as described above and all transcripts were amplified with an efficiency around 94% (E = 1.94) and *R*^2^ = 0.9963, without significant differences among them.

#### Statistical analysis

Expression levels of each transcript were compared among the five groups generated according to the number of copies of the CNV per individual (from 2 to 6) or between low and high CNV copies using Kruskal-Wallis and Mann-Whitney U non-parametric tests, respectively. In both cases normality was previously rejected using the Kolmogorov-Smirnov test. To estimate the relationship among the number of copies of the CNV and the expression levels of each transcript, a linear regression analysis was performed and its correlation was confirmed using the Spearman’s rank correlation coefficient (ρ), threshold set at *P* < 0.05 in all tests. Statistical analysis was performed using SPSS Statistics Version 22.0 software (IBM Corp. Released 2013. Armonk, NY).

#### Ethics statement.

This study was approved by the local Ethics Committee and informed consent was obtained from all adult subjects, children, and their parents according to the Helsinki declaration.

## Additional Information

**How to cite this article**: Cabana-Domínguez, J. *et al.* A highly polymorphic copy number variant in the *NSF* gene is associated with cocaine dependence. *Sci. Rep.* **6**, 31033; doi: 10.1038/srep31033 (2016).

## Supplementary Material

Supplementary Information

## Figures and Tables

**Table 1:** Association study of the CNV in the *NSF* gene in cocaine-dependent Spanish patients.

Genotype	Discovery		Replication		Pooled analysis^1^	
	Controls N (%)	Cases N (%)	Controls N (%)	Cases N (%)	Controls N (%)	Cases N (%)
Low copy*	226 (63.5)	260 (72.4)	349 (61.3)	352 (69.3)	575 (62.2)	612 (70.6)
High copy**	130 (36.5)	99 (27.6)	220 (38.7)	156 (30.7)	350 (37.8)	255 (29.4)
SUM	356	359	569	508	925	867
p-value	**0.01287**		**0.00713**		**0.00017**	
(OR −95% CI)	1.51 [1.10–2.07]&		1.42 [1.10–1.83]&		1.46 [1.19–1.78]&	

In bold: significant p-values.

^1^Discovery + replication samples.

^*^Low copy genotype include genotypes 2 and 3.

^**^High copy genotype include genotypes 4, 5 and 6.

^&^When OR < 1. the inverted score is shown.

**Table 2:** Haplotype frequencies in the *NSF* gene, including SNPs and CNV.

		rs183211-rs17698176		
		CC	GT	AT
**Copy number**	**1**	0.0102	0.4984	0.0491
	**2**	0.0632	0.0449	0.2312
	**3**	0.0765	0.0046	0.0220
