# Supplementary material for: A Highly Polymorphic Copy Number Variant in the NSF Gene is Associated with Cocaine Dependence
Source: Sci Rep. 2016 Aug 8;6:31033. doi: 10.1038/srep31033 (PMC4976312; doi:10.1038/srep31033)
Supplement: Supplementary Information [file srep31033-s1.pdf]

A HIGHLY POLYMORPHIC COPY NUMBER VARIANT IN THE *NSF* GENE IS ASSOCIATED WITH COCAINE DEPENDENCE

Judit Cabana-Domínguez[1,2,3], Carlos Roncero[4,5,6,7], Lara Grau-López[4,5,6,7], Laia Rodríguez-Cintas[5,6], Carmen Barral[4,5,6,7], Alfonso C. Abad[4,5], Galina Erikson[8], Nathan E. Wineinger[8], Bàrbara Torrico[1,2,3], Concepció Arenas[9], Miquel Casas[4,6,7], Marta Ribasés [4,7,10], Bru Cormand*[1,2,3 &], Noèlia Fernàndez-Castillo*[1,2,3 &]

Supplementary Table S1. Association study of CNV in the NSF gene in cocaine dependent Spanish patients.

	Discovery		Replication		Pooled analysis[1]	
**Number of copies**	**Controls N (%)**	**Cases N (%)**	**Controls N (%)**	**Cases N (%)**	**Controls N (%)**	**Cases N (%)**
**2**	92 (25.8)	118 (32.9)	150 (26.4)	152 (29.9)	242 (26.2)	270 (31.5)
**3**	134 (37.6)	141 (39.3)	199 (35.0)	201 (39.6)	333 (36.0)	342 (39.9)
**4**	87 (24.4)	75 (20.9)	134 (23.6)	103 (20.3)	221 (23.9)	178 (20.7)
**5**	33 (9.3)	22 (6.1)	62 (10.9)	37 (7.3)	95 (10.3)	59 (6.9)
**6**	10 (2.8)	3 (0.8)	24 (4.2)	15 (3)	34 (3.7)	18 (2.1)
**SUM**	356	359	569	508	925	867
**p-value**	**0.03696**		0.06021		**0.00134**	

In bold: significant p-values. [1] Discovery + replication samples.

Supplementary Table S2. Haplotype frequencies.

rs183211	CNV	Frequencies
A	1	0.045
A	2	0.235
A	3	0.022
G	1	0.515
G	2	0.101
G	3	0.083

Supplementary Table S3. Primer sequences used in qRT-PCR assays.

Gene	Region or transcript		Nucleotide sequence (5'→3')
ALB	Intron 12	Fw	TTGTGGGCTGTAATCATCG
		Rv	TGCTGGTTCTCTTTCACTGAC
GAPDH	GAPDH_001	Fw	AGCCACATCGCTCAGACAC
		Rv	GCCCAATACGACCAAATCC
HPRT1	HPRT1_001	Fw	TGATAGATCCATTCCTATGACTGTAGA
		Rv	CAAGACATTCTTTCCAGTTAAAGTTG
NSF	Exon 2	Fw	GGCAAGATGTCCTACAGATGAA
		Rv	CGAATGCCACTTAGCAGGTTA
	Exon 13	Fw	GCAAGTGACGAGAGGAGAC
		Rv	TGGCACTTACTTTTCGCCTG
	Intron 13	Fw	GAGGGTTTGAGTCTGGTGGA
		Rv	AGCCTGGAAAGCAAAACAACT
	NSF_001 + NSFP1_001	Fw	CCCCCAGGTTGTGGTAAGAC
		Rv	ACTGTTAGCACCAAGCCTCC
	NSF_001	Fw	GCTGATCGAGATGTCCCTAC
		Rv	CTTCAGGCAAAGAGAATACCAG
	NSF_002	Fw	ACTGAAGACACATCCATCGGT
		Rv	TACCATAACTATTTCCTTCCC
	NSF_003	Fw	GCCACAGGAGAGCAGC
		Rv	CCTTGCTTGGTCTCTCGG
